# Supplementary material for: Evaluation of Immunogenicity and Clinical Protection of SARS-CoV-2 S1 and N Antigens in Syrian Golden Hamster
Source: Vaccines (Basel). 2022 Nov 24;10(12):1996. doi: 10.3390/vaccines10121996 (PMC9781188; doi:10.3390/vaccines10121996)
Supplement: Supplementary file 1 [file vaccines-10-01996-s001.zip › vaccines-2009342-supplementary.pdf]

Supplementary

Evaluation of immunogenicity and clinical protection of SARS-CoV-2 S1 and N antigens in Syrian golden hamster

Zhenye Niu, Xueqi Li, Yang Gao, Lichun Wang, Shengtao Fan, Xingli Xu, Guorun Jiang, Pingfang Cui, Dandan Li, Yun Liao, Li Yu, Heng Zhao, Ying Zhang*, Qihan Li*

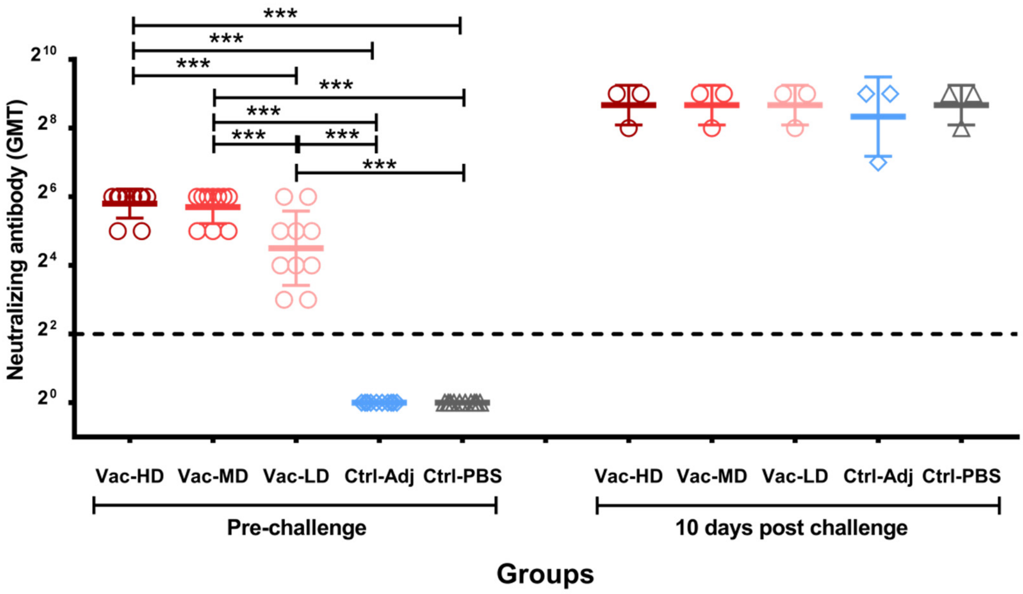

Supplementary Figure S1. Neutralizing antibody response induced by experimental inactivated vaccines administered via the IM route. The hamsters were immunized with two doses of experimental inactivated vaccines at an interval of 14 days via the IM route. The titers of neutralizing antibodies were detected at 14 days after booster immunization (pre-challenge) and at 10 days after challenge (10 days post challenge). Vac-HD: high-dose vaccine group (150 U/dose); Vac-MD: medium-dose vaccine group (100 U/dose); Vac-LD: low-dose vaccine group (50 U/dose); Ctrl-Adj: adjuvant control group; Ctrl-PBS: negative control group. The dotted line in the neutralizing antibody graph represents the positive threshold (neutralizing antibody titer of 4). ***, $p<0.001$. ($n=10$ per group)

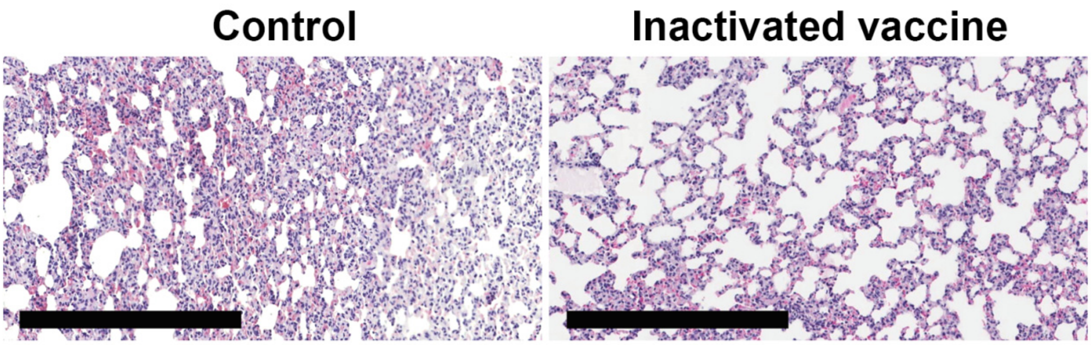

Supplementary Figure S2. Pathological damage to the lungs in the hamsters immunized with inactivated vaccines via the IM routes during viral challenge.

H&E-stained lung sections collected from virus-challenged hamsters at 10 days after challenge (scale bars, 0.4 mm).
